# Physician Survey on the Management of Pulmonary Embolism in Pregnancy: Need for Guidance and Training

**DOI:** 10.1002/jha2.70113

**Published:** 2025-07-28

**Authors:** Giulia Simini, Richard Buka, Daniel Kajita, Omar Mukhlif, HaemSTAR collaborators, Gill Swallow, Pip Nicolson, Sajida Kazi

**Affiliations:** ^1^ Department of Haematology Royal Free Hospital London UK; ^2^ Department of Haematology Cancer Institute University College London London UK; ^3^ Institute of Cardiovascular Sciences University of Birmingham London UK; ^4^ Department of Haematology Nottingham University Hospitals NHS Trust Nottingham UK; ^5^ Department of Haematology University Hospitals of Leicester Leicester UK; ^6^ Department of Haematology Newcastle Upon Tyne Hospitals NHS foundation Trust Newcastle UK

1

The Confidential Enquiry into Maternal Deaths has consistently identified thrombosis and thromboembolism as the leading causes of direct maternal mortality in the UK and Ireland between 2013 and 2023 [1]. Among these, high‐risk pulmonary embolism (PE)—previously referred to as *massive PE*—is characterized by hemodynamic instability due to obstruction of the pulmonary arterial circulation and represents a particularly critical scenario, with mortality rates as high as 37% [2]. Intermediate‐high risk PE, formerly known as *submassive PE*, also carries a significant mortality risk of up to 10% [3].

Systemic thrombolysis remains one of the most effective treatments for high‐risk PE [4, 5]. However, in pregnancy, its use is fraught with significant risks, most notably the potential for life‐threatening uterine hemorrhage [6]. In recent years, alternative advanced reperfusion options, including catheter‐directed thrombolysis (CDT), endovascular thrombectomy, and surgical thrombectomy have emerged as alternative options [7, 8, 9, 10]. While these approaches show great promise, they lack validation from large randomized controlled trials [11]. Approaches to management of intermediate risk PE are less clearly defined [12].

Efforts to bridge this evidence gap are underway: the International Society on Thrombosis and Haemostasis launched a global registry in 2019 to collect data on high‐risk PE management during pregnancy and puerperium [13]. Registries are invaluable for identifying trends and improving understanding but face challenges like differing legal‐ethical frameworks across countries. This forces clinicians to rely on single‐center expertise and institutional protocols, leading to inconsistent care. Pulmonary Embolism Response Teams (PERTs) are developing frameworks designed to facilitate a coordinated multidisciplinary approach to decision‐making in PE cases [14]. However, their national distribution is unbalanced. Addressing these inconsistencies on a national scale is essential to establish standardized approaches, gather robust data, and support the development of randomized clinical trials in this under‐researched area.

To explore current practices and identify potential gaps in care, we conducted a national survey targeting clinicians involved in the management of PE in pregnancy across the UK and Ireland. The survey was distributed through the HaemSTAR network, a trainee‐led organization that facilitates nationwide research and audits. HaemSTAR's decentralized network of regional representatives enabled targeted, and rapid data collection from diverse locations. The survey was administered electronically via Google Forms, following a process of informal validation through a two‐stage feedback process. First, the survey pilot was distributed to the HaemSTAR committee for internal review, during which question clarity, relevance, and structure were assessed. Based on this feedback, iterative refinements were made. The revised version was then shared with three independent consultant physicians to obtain external expert feedback. The questionnaire included multiple‐choice, multiple‐answer (checkbox), Likert scale (5‐point), ranking, and free‐text questions. These formats were used to assess institutional practices, clinical protocols, clinician confidence, perceived utility of evidence sources, and decision‐making factors related to PE management in pregnancy.

The survey, conducted between January and March 2024, garnered 70 responses. Participants were predominantly highly experienced specialists, with 87% reporting over 10 years of practice. The majority were based in tertiary centers or teaching hospitals and represented a range of specialties, including hematology, obstetrics, acute medicine, radiology, and intensive care. Geographically, responses were well‐distributed across England, with fair representation from Scotland, Wales, Northern Ireland, and Ireland (Table 1).

**TABLE 1:** Summary of respondents’ geographical distribution, background and their access to advanced therapies, PERT and local trust guidelines.

Respondents (N = 70)	N (%)
**Geographical distribution**	
England	57 (81)
Scotland	4 (6)
Wales	3 (4)
Northern Ireland	2 (3)
Ireland	4 (6)
**Years of experience**	
>15	42 (60)
11–15	19 (27)
6–10	8 (11)
<5	1 (12)
**Type of Hospital**	
Tertiary center	48 (68)
District General Hospital	22 (32)
**Specialties**	
Hematology	52 (74)
Obstetrics & Gynecology	3 (4)
Critical Care	2 (3)
Internal Medicine	7 (10)
Other	6 (9)
**Availability of advanced therapies**	
Catheter directed thrombolysis	57 (81)
Surgical Thrombectomy	47 (67)
ECMO	52 (74)
**PERT availability**	
Yes, always available	8 (11)
Yes, available during restricted hours	16 (23)
Not available	33 (47)
Not sure/Never contacted	13 (19)
**Received dedicated training in**	
Management of PE in pregnancy	20 (28)
Systemic thrombolysis	14 (72)

Abbreviations: ECMO, extracorporeal membrane oxygenation; PERT, pulmonary embolism response team.

Advanced treatment modalities access was present in most centers: 74% had CDT 52% had extracorporeal membrane oxygenation (ECMO), and 60% had surgical thrombectomy either on site or less than one hour away (Table 1). Despite this, the availability of local trust guidelines was inconsistent. While most centers had local thrombolysis protocols, there was a lack of local guidelines on alternative reperfusion therapy. Only 50% reported dedicated guidelines on CDT and 17% reported no specific protocols for managing pregnancy‐related PE.

The survey also revealed limited training for advanced PE management in pregnancy. Only 38% of respondents reported having received focused training on pregnancy‐related PE, and just 22% had undergone training in systemic thrombolysis. Although this is quite a rare event, bespoke training is still needed as there is limited chance to learn from experience.

Looking at our respondents’ cohort, 46% reported that their centers did not have access to a PERT. These were also not uniformly available across the UK, with the majority being in London (60%), and the remainder distributed among East Midlands (15%) Yorkshire (10%), West Midlands (5%), South Peninsula (5%) and North‐West (5%). In addition, nearly 20% of respondents were unsure whether their hospital had a PERT. A recent meta‐analysis from Hobohm et al. has highlighted that PERT involvement contributed to shorter inpatient time and more frequent use of advanced therapies [14]. Some studies have even shown a lower mortality in groups where PERTs were implemented, although evidence is heterogeneous [15].

Respondents’ opinion of treatment varied, as summarized in Figure 1. While 70% considered thrombolysis appropriate for high‐risk PE, only 20% felt it was suitable for intermediate‐risk PE. In high‐risk cases, CDT was viewed as useful or extremely useful by 72% of respondents, while 11% considered it rarely useful and the reminder was unsure. In intermediate‐high risk cases, just under 50% of respondents considered CDT a useful option and an equal proportion indicated this is rarely useful/not useful at all. Although thrombectomy was considered more useful in high‐risk PE by more than half of the cohort, only a third viewed useful in intermediate‐high risk cases. Confidence in treatment indications also remained variable. 29% of respondents reported uncertainty regarding the indications for thrombolysis in pregnancy, and 36% were unsure about the use of CDT or thrombectomy in this context. A recent review of CDT usage trends in Germany (2002–2020), which included pregnant patients, found a steady increase in its use, with lower mortality and fewer serious adverse events. However, only 0.2% of high‐risk PE cases were treated with this approach. Notably, CDT was preferentially used in younger patients and those of male sex [16].

**FIGURE 1:**
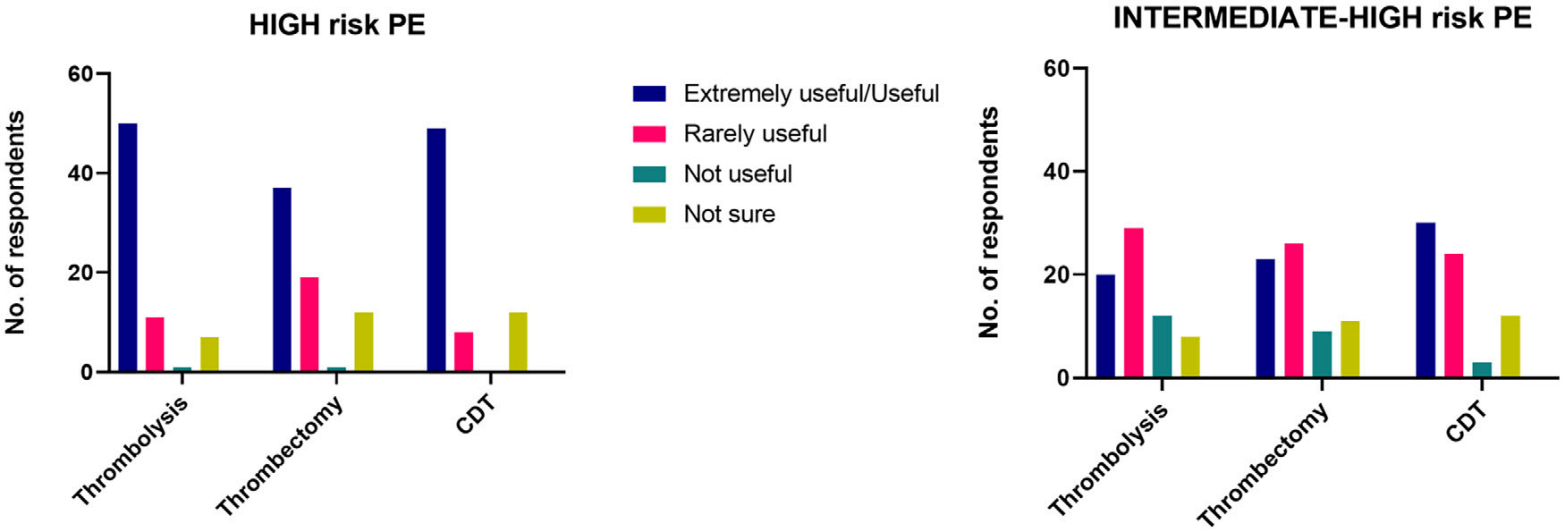
Physicians' perceptions of the usefulness of advanced treatments for high and intermediate‐high risk pulmonary embolism (PE). CDT, catheter‐directed thrombolysis.

Next, we investigated which individuals or roles are authorized to decide on systemic thrombolysis. 47% indicated that one consultant holds this responsibility, while 32% reported that decisions require two separate consultants. Specialty registrars were responsible in 19% of cases, with smaller proportions involving PERT teams or uncertainty about the protocol. This highlights the variability in decision‐making practices across hospitals.

Limitations of the questionnaire include a relatively small sample size, a predominance of responses from tertiary centers compared to district general hospitals, and the inherent potential for self‐reporting bias.

In conclusion, this survey highlights key challenges in managing PE in pregnancy and suggests potential strategies to address them. Firstly, studying the real‐world management of intermediate‐ and high‐risk PE in pregnancy is important to provide baseline, patient level data to inform quality improvement programs. In direct response to these findings, the MATRON project (Maternal Audit on ThRombosis Outcome and DecisioN) is being conducted as a national, multi‐center retrospective audit, led by HaemSTAR in collaboration with the UK obstetrics and gynaecology research network UKARCOG. The project is collecting real‐world data on the management and outcomes of high‐risk and intermediate‐high‐risk PE in pregnant and postpartum patients across the UK. Using a flash‐mob model of data collection through national trainee research networks, MATRON aims to address key evidence gaps and inform the development of pregnancy‐specific clinical guidance. Secondly, integrating standardized training curricula, including dedicated obstetric hematology programs, could improve clinician preparedness. Thirdly, expanding access to advanced therapies and PERTs could help optimize outcomes and ensure equitable care. In addition, developing and disseminating clear, evidence‐based guidelines is essential to reduce practice variability and enhance patient care. Finally, a coordinated approach—through multicenter trials, robust national guidelines, and improved training–is crucial for optimizing management and outcomes.

## Author contributions

Giulia Simini conceived the study, analyzed the data, wrote and revised the manuscript. Sajida Kazi, Richard Buka, Pip Nicolson, and Gill Swallow revised and edited the manuscript. Daniel Kajita, Omar Mukhlif, and HaemSTAR collaborators distributed and promoted the survey. All authors have reviewed and approved the final manuscript.

## Conflicts of Interest

Giulia Simini is funded by BioMarin Pharmaceutical Inc. for research outside this project.Pip Nicolson received grant funding from Sanofi S.A., Rigel Pharmaceuticals and Novartis for research outside this project. Pip Nicolson received speaker fees from Sobi and Takeda Pharmaceuticals. Richard Buka received funding from AstraZeneca for research outside this project, honoraria from Bayer, Sobi, Sanofi S.A. and Viatris and royalties from Karger. The authors have no other conflicts of interest to declare.

## Ethics Statement

The authors have nothing to report.

## Clinical Trial Registration (including trial number)

The authors have confirmed clinical trial registration is not needed for this submission.

## Data Availability

The data that support the findings of this study are available from the corresponding author upon reasonable request.
